# Involvement of corneal epithelial cells in the Th17 response in an in vitro bacterial inflammation model

**Published:** 2013-01-17

**Authors:** Isabel Arranz-Valsero, Ute Schulze, Laura Contreras-Ruiz, Laura García-Posadas, Antonio López-García, Friedrich Paulsen, Yolanda Diebold

**Affiliations:** 1 Ocular Surface Group-IOBA, University of Valladolid, Valladolid, Spain; 2 CIBER-BBN (Biomedical Research Networking Center in Bioengineering, Biomaterials, and Nanomedicine), Spain; 3 Department of Anatomy and Cell Biology, Martin Luther University Halle/Wittenberg, Halle/Saale, Germany; 4 Department of Anatomy II, Friedrich Alexander University Erlangen/Nuremberg, Erlangen, Germany

## Abstract

**Purpose:**

*Staphylococcus aureus* (SA) and *Pseudomonas aeruginosa* (PA) are frequent causes of bacterial keratitis, an inflammatory process that can lead to vision loss. We used a human corneal epithelial (HCE) cell line to study the Th17 inflammatory pathway, including interleukin (IL-) 6, IL-17, and associated receptors, in response to stimulation by SA and PA culture supernatants.

**Methods:**

Cells of the HCE cell line were exposed to either SA or PA supernatants in dilutions of 1:100 or 1:50, or to human recombinant IL-17A (20 ng/ml). Cell culture supernatants were collected at 6, 24, and 72 h, and protein and RNA were isolated. Expression of cytokine (IL-6, IL-17A), receptor (sIL-6R, IL-17RA), and mediator (soluble glycoprotein [sgp] 130, MIP3α) proteins and mRNAs were determined with enzyme-linked immunosorbent assay, immunohistochemistry, western blotting, and real-time, reverse-transcription quantitative PCR. In addition, IL-17RA was localized by transmission electron microscopy after immunogold labeling.

**Results:**

Basal secretion of IL-6 and IL-17A by HCE cells occurred in a time-dependent manner. Expression of IL-6 was significantly enhanced by SA stimulation, but not by PA stimulation. IL-6 mRNA expression was higher in the control and SA-stimulated cells at 6 and 24 h, but not at 72 h. In the PA-stimulated cells, mRNA levels were significantly lower than the controls at 6 and 24 h. Expression of sIL-6R was not altered by SA or PA supernatants, but sgp130 expression was greater than controls at 6 h, less than controls at 24 h, and the same as controls at 72 h. HCE cells secreted IL-17A in a time-dependent manner that was not altered by stimulation; however, the IL-17A mRNA levels were lower than those of the controls at 6 h. With immunohistochemistry, IL-17RA was localized in perinuclear vesicles and in the cytosol and membranes of HCE cells. IL-17RA was also present in the epithelial cells from human ocular surface tissues. As quantified with western blotting, expression of IL-17RA was unchanged in HCE cells stimulated by SA or PA supernatants.

**Conclusions:**

HCE cells react to bacterial inflammation by enhancing the secretion of IL-6 and by regulating the proinflammatory response with differential secretion of sgp130. Under normal conditions, HCE cells and ocular surface tissues express IL-17RA. Additionally, HCE cells express IL-17RA after bacterial stimulation. All of these molecules are involved in the Th17 differentiation pathway, suggesting that corneal epithelial cells may act as indirect participants in the Th17 signaling pathway.

## Introduction

*Staphylococcus aureus* (SA) and *Pseudomonas aeruginosa* (PA) are frequent causes of bacterial keratitis, an inflammatory process that can lead to vision loss. Both pathogens are usually considered extracellular bacteria, growing as biofilms on mucous membranes. However, the pathogens can sometimes invade corneal epithelial cells and cause inflammation [1-3]. In some cases, once the infection is controlled, host defense mechanisms may maintain an activated status and contribute to initiating a chronic inflammatory process. For instance, bacterial lipopolysaccharide can trigger intracellular signaling cascades via the Toll-like receptor 4. This signal rapidly induces inflammatory cytokine production that initiates various overlapping immune responses [4]. Among the different immune responses, the Th17 pathway is the main pathway activated during infection with extracellular pathogens [5,6]. Cytokines secreted by immune cells or by the infected cells, among other environmental and genetic factors, are the main inducers of Th17 pathway activation [7].

Interleukin (IL)-6 is a multifunctional cytokine involved in a broad variety of ocular inflammatory conditions. For instance, IL-6 has a protective role during corneal infection with PA [8]. IL-6 is also one of the major cytokines responsible for differentiating T helper lymphocytes into Th17 cells [9]. IL-6 signal transduction needs a specific transmembrane receptor (IL-6R) and activation of the transmembrane glycoprotein (gp) 130, leading to their dimerization and hexameric complex formation [10]. Although IL-6R expression is mainly limited to hepatocytes and some leukocytes [11], IL-6 is expressed in cytokine-treated human corneal epithelial and normal human conjunctival cell lines [12]. Nonetheless, the immune system can increase the number of potential IL-6 target cells with the IL-6 trans-signaling pathway: IL-6 binds the soluble form of IL-6R (sIL-6R) [13] and transmits the signal through the transmembrane gp130. The ability of ocular surface cells to produce sIL-6R has been reported [14-16], but involvement in bacterial inflammatory conditions remains unknown.

IL-17 is the hallmark cytokine of the recently described Th17 cells [17]. Six isoforms are known (IL-17A–F), and expression varies depending on cell type, tissue, and disease [18]. Some innate sources of IL-17, such as natural killer and myeloid cells, have been reported [19] and are thought to act before adaptive immunity takes place. IL-17A acts as the main cytokine responsible for initiating innate responses against infection by stimulating the production of cytokines, neutrophil chemoattractants, and antimicrobial peptides. IL-17A-producing cells have been identified in the mid-peripheral cornea in a mouse model of dry eye disease [20]. This cytokine is also expressed in corneas from patients with herpetic stromal keratitis [21]. However, IL-17A production is usually linked to leukocytes, while IL-17C is linked to epithelial cell host defense, acting in an autocrine manner [22]. To the best of our knowledge, IL-17A production by corneal epithelial cells has not been described.

IL-17A signal transduction needs at least two receptors, among the five receptors described (IL-17RA–E), but the highest affinity appears with the binding of IL-17A to IL-17RA [23,24]. IL-17RA is expressed in the majority of human cell membranes [25], e.g., human leukocytes [26,27], human bronchial epithelial cells [28], and human corneal fibroblasts [21], but little is known about IL-17RA within the epithelial cells of the ocular surface. A protective role against bacterial inflammation has been postulated for IL-17 [29]. Among other roles, this cytokine is able to increase MIP3α, a chemoattractant protein with antimicrobial properties, as demonstrated in airway epithelial cells in vitro [30]. However, IL-17 and other Th17-related molecules may also be responsible for the pathogenesis of disease [17,31], as observed in IL-17-deficient mice, which are susceptible to inflammatory autoimmune diseases in a rodent model of collagen-induced arthritis [32]. Understanding the mechanisms of action of Th17-related cytokines may be of great value to enhance the knowledge of severe or chronic ocular inflammatory diseases caused by bacterial infections. Although animal models of bacterial keratitis have been described [33], an in vitro model can be helpful to better understand the contribution of corneal epithelial cells to the Th17 response during the infection. The aim of this work was to determine whether corneal epithelial cells secrete cytokines and other effectors of the Th17 pathway in response to molecular stimuli derived from bacteria.

## Methods

### Materials

Tissue culture plastics, including multichamber Permanox slides were obtained from Nunc (Roskilde, Denmark). Culture medium and supplements, Alexa Fluor-488 donkey antirabbit secondary antibody (Ab) for immunofluorescence analysis, TRIzol reagent, RNase inhibitor, and SuperScript VILO cDNA Synthesis Kit were purchased from Invitrogen-Gibco (Inchinnan, Scotland). Donkey serum, Laemmli Sample Buffer, and Tween-20 were from Sigma-Aldrich (St. Louis, MO). Materials for bacterial culture included tryptone soy broth from Oxoid (Basingstoke, England), sheep blood from Heipha (Eppelheim, Germany), and filters from Millipore (Eschborn, Germany). Human recombinant IL-17A was from Prospec (Ness-Ziona, Israel). Enzyme-linked immunosorbent assay (ELISA) kits for IL-6, IL-17A, and MIP3α were from RayBiotech (Norcross, GA), and those for soluble glycoprotein (sgp) 130 and sIL-6R were from Diaclone (Besançon, France). The bicinchoninic acid (BCA) protein quantification method was from Pierce (Rockford, IL). Materials used for sodium dodecyl sulfate–polyacrylamide gel electrophoresis (SDS–PAGE) and western blotting, including analysis software, were purchased from Bio-Rad (Hercules, CA). Low fat milk powder and antibodies for western blotting were obtained from Santa Cruz Biotechnology (Santa Cruz, CA), except donkey antimouse affine pure peroxidase-conjugated immunoglobulin, which came from Jackson ImmunoResearch Laboratories (West Grove, PA). Propidium iodide was from Molecular Probes (Eugene, OR). Reagent for silver enhancement R-Gent SE-EM was from Aurion (Wageningen, Netherlands), and Epon resin Glycidether 100 from Carl Roth GmbH + Co KG (Karlsruhe, Germany). VECTASHIELD medium and VECTASTAIN ABC kits were from Vector Laboratories (Burlingame, CA). Reagents for immunohistochemistry were from Dako (Glostrup, Denmark). RNase-free DNase I was from Boehringer (Mannheim, Germany). Validated primers PPH00537B and PPH00983A were from SABiosciences (Frederick, MD), and 2X SYBR Green Real-time PCR Master Mix was from Qiagen (Hilden, Germany).

### Human corneal epithelial cell line

A human corneal epithelial (HCE) cell line [34] was cultured in Dulbecco’s Modified Eagle Medium (DMEM)/F-12 + GlutaMAX-I supplemented with 10% fetal bovine serum. Cells were maintained at 37 °C in a 5% CO_2_ atmosphere, and the culture medium was changed every other day. Daily observations were done with phase contrast microscopy.

### Production of bacterial supernatants

Bacterial supernatants from SA strain SA113 (ATCC 35,556) and PA strain PA01 (ATCC 15,692) were obtained as previously described by Paulsen et al. [35]. Briefly, laboratory strains of the respective bacteria were grown overnight at 37 °C by shaking in tryptone soy broth. Thereafter, tenfold bacterial dilutions were plated on Columbia agar supplemented with 10% sheep blood overnight at 37 °C. Bacteria at 5×10^7^ cfu/ml were centrifuged twice at 2,000 ×g for 30 min, and the supernatants filtered twice using filters impermeable to bacteria (0.22 µm pore size). The sterility of the supernatant aliquots was tested with overnight incubation on agar.

### Stimulation of human corneal epithelial cells with bacterial supernatants or interleukin-17A

A total of 3×10^5^ HCE cells/dish were seeded in individual 8.8 cm^2^ area Petri dishes and cultured for 72 h. The cells were then maintained for 24 h in non-supplemented culture medium. After that, the cells were exposed to either SA or PA supernatants (1:100 and 1:50 dilution), or to IL-17A (20 ng/ml in serum-free DMEM). Cell culture supernatants were collected at 6, 24, and 72 h and used for ELISA. The concentration of the secreted cytokines (IL-6, IL-17A), soluble receptor (sIL-6R), and mediators (sgp130, MIP3α) in cell supernatants was measured according to the manufacturer’s instructions. Cell lysates were used for protein determination by SDS–PAGE and western blotting or for mRNA determination with real-time, reverse-transcription quantitative polymerase chain reaction (qPCR). All samples were run in duplicate, in three independent experiments.

### Cytotoxicity assay

Cytotoxicity of bacterial supernatants was measured with the tetrazolium salt, 2,3-bis-(2-methoxy-4-nitro-5-sulfophenyl)-2H-tetrazolium-5-carboxanilide (XTT)-based, colorimetric assay. Stimulation of HCE cells with bacterial supernatants was performed for 6, 24, and 72 h as described above. After stimulation, 100 μl of the XTT working solution (20% of XTT stock solution [1 mg/ml] in Roswell Park Memorial Institute (RPMI) without phenol red culture medium) was added to each well. Cells were incubated at 37 °C for 16 h, and then the optical density was measured using a microplate multireader at 450 nm (reference wavelength: 620 nm). Three different experiments were performed for each condition. Cell viability was calculated as a percentage of viable cells in the treated group versus an untreated control by employing the following equation:

cell viability (%)=ODtreatment/ODcontrolx100

### RNA preparation and cDNA synthesis

Cell lysates were prepared from HCE cells using TRIzol reagent, and RNA was isolated using chloroform. Crude RNA was purified with isopropanol and repeated ethanol precipitation. Contaminating DNA was destroyed by digestion with RNase-free DNase I and RNase inhibitor (30 min at 37 °C). Inactivation of the enzyme was done by heating at 65 °C for 10 min. A total of 2 µg of purified RNA was used for each reaction of reverse transcription with the SuperScript VILO cDNA Synthesis Kit.

### Quantification of interleukin-6, interleukin-17A, and interleukin-17RA mRNA expression levels with quantitative polymerase chain reaction

The IL-6, IL-17A, and IL-17RA mRNA levels were quantified with qPCR. Primer pair oligonucleotides were designed for IL-6 mRNA and for 18S rRNA that was used as a reference gene. Validated primers were bought for IL-17A and IL-17RA. Primer sequences or references and the amplification procedure for each primer pair are shown in Table 1. The PCR reaction was performed in a volume of 20 μl using 10 µl of 2X SYBR Green Real-time PCR Master Mix, 0.4 µg of synthesized cDNA, and the specific primer pair at 5 nM, in an Applied Biosystems 7500 Real-Time PCR System (Foster City, CA). The fluorescence signal was digitally collected after each cycle of 72 °C. Relative quantification of the signals was done by normalizing the signal of the cytokines with the 18S rRNA signal. The semiquantitative comparative ΔΔCt method was used to analyze the relative gene expression of each sample. Controls included no-template controls and no-reverse-transcription controls. In addition, after each PCR, samples were subjected to a temperature ramp with continuous fluorescence monitoring for melting curve analysis to verify that the fluorescence signal was due to the amplification of a single fragment.

**Table 1:** Gene primer pairs and amplification procedures.

Gene	Primer sequence (5’-3’)	Amplification procedure			Length (bp)
		Cycles	T (°C)	Time (s)	
IL-6	F: GACAGCCACTCACCTCTTCA R: CATCCATCTTTTTCAGCCATC	1	94	180	171
		36	94	20	
			59.1	30	
			72	30	
IL-17A	Reference: PPH00537B Unknown sequence	1	50	120	Unknown
		1	95	600	
		40	95	15	
			60	60	
IL-17RA	Reference: PPH00983A Unknown sequence	1	50	120	Unknown
		1	95	600	
		40	95	15	
			60	60	
18S	F: ACTCAACAGGGGAAACCTCAGC R: CGCTCCACCAACTAAGAACGG	1	50	120	250
		1	94	180	
		40	94	20	
			59	33	
			72	40	

### Interleukin-17RA detection in human ocular surface tissues with immunohistochemistry

For immunohistochemistry analysis, human tissue samples were obtained from bodies donated by testament to the Department of Anatomy and Cell Biology, Martin Luther University Halle-Wittenberg, Germany, for research or teaching purposes, in accordance with German and European Union laws. In addition, the study had the approval of the institutional review boards of the University of Valladolid and the University of Halle-Wittenberg. Samples of human cornea (n=6), limbus (n=4), and conjunctiva (n=6) were fixed in 4% formalin, embedded in paraffin, sectioned (7 µm), and deparaffinized. HCE cells (either exposed to IL-17A or unexposed) were seeded as in the immunofluorescence assays and used as controls. Samples were fixed in 4% paraformaldehyde and 0.1% glutaraldehyde for 7 min. They were then incubated for 10 min with Enzyme Block blocking solution and 1 h with goat serum. Then, non-specific binding was inhibited with 5% goat serum in Tris-buffered saline with 0.05% Tween 20 (TBS-T). Primary polyclonal rabbit antihuman IL-17RA Ab was used at 1:100 dilution in blocking solution, overnight at 4 °C. The biotinylated goat antimouse secondary Ab was added at 1:100 in blocking solution and incubated at room temperature (RT) for 30 min. Samples were incubated with the mix of the VECTASTAIN ABC kit for 30 min and then with 2% diaminobenzidine for 5 min. Nuclei were stained by quickly dipping the slides in hematoxylin.

### Interleukin-17RA detection in human corneal epithelial cells with electrophoresis and western blotting

HCE cells were homogenized in 1% Triton X-100 in PBS (KCl: 200 mg/l; KH_2_PO_4_: 200 mg/l; NaCl: 8,000 mg/l; Na_2_HPO_4_.7H_2_O 2160 mg/l). After homogenization, the samples were incubated for 30 min on ice and then centrifuged at 15,000 ×g for 30 min at 4 °C. Total cell protein in the resulting supernatant was measured with the BCA method, which was compatible with the buffer used for homogenization. Bovine serum albumin (BSA) was used as the standard.

Protein in cell lysates were mixed 1:1 with Laemmli Sample Buffer and boiled for 5 min. Equal amounts of proteins (10 μg/lane) were resolved with SDS–PAGE on 8% acrylamide gels (15 min at 70 V and 1 h 30 min at 110 V). Proteins were then transferred to nitrocellulose membranes (1.5 h at 350 mA). Nonspecific binding was blocked in TBS-T and supplemented with 4% donkey serum and 5% non-fat milk (blocking buffer) for 1 h at RT. Membranes were then incubated with primary Abs diluted in blocking buffer, washed three times with TBS-T, and incubated with corresponding secondary Abs diluted in TBS-T (Table 2). Membranes were incubated with Immun-Star Horseradish Peroxidase Buffer and Immun-Star Horseradish Peroxidase Luminol/Enhancer (Bio-Rad), according to the manufacturer’s protocol. Immunoreactive bands were visualized with the chemiluminescence method (ChemiDoc XRS; Bio-Rad, Hercules, CA), and images were analyzed using the Quantity One software. Proteins in the cell lysates of the leukemia chronic myelogenous K562 cell line were used as controls to assess the specificity of the anti-IL-17RA Ab.

**Table 2:** Antibodies (Ab) and conditions for immunodetection of indicated molecules.

Antibodies		Reference	Dilution	Time of incubation	Temperature (°C)
IL-17RA					
1^st^ Ab	Rabbit anti-human	sc-30175	WB 1:200	Overnight	4
			IMF 1:50		
2^nd^ Ab	Goat anti-rabbit	sc-2004	WB 1:2000	1 h	RT
	Donkey anti-rabbit	A-21206	IMF 1:100		

α-Actinin					
1^st^ Ab	Mouse anti-human	sc-17829	1:500	Overnight	4
2^nd^ Ab	Donkey anti-mouse	715–035–150	1:5000	1 h	RT

### Interleukin-17RA detection in human corneal epithelial cells with immunofluorescence

HCE cells were seeded on eight-well multichamber Permanox slides (3×10^4^ cells/well), cultured for 72 h, and exposed to SA and PA supernatants up to 72 h. At the end of each experiment, cells were fixed for 7 min in cold methanol. Nonspecific binding was inhibited with 4% donkey serum in PBS (blocking solution). Slides were incubated with primary Ab diluted in blocking solution, washed three times, and incubated with secondary Ab in PBS (Table 2). Nuclei were stained with propidium iodide 1:10,000 in PBS. Slides were mounted in VECTASHIELD medium, and the preparations were viewed in an epifluorescence microscope (Leica Microsystems DMI 6000B, Wetzlar, Germany). Negative controls included the omission of primary Ab. All samples were run in duplicate, in three independent experiments.

### Interleukin-17RA detection in human corneal epithelial cells with transmission electron microscopy after immunogold labeling

Cultured HCE cells were grown on plastic coverslips, exposed to recombinant IL-17A (20 ng/ml), and fixed after confluence in 4% (vol/vol) paraformaldehyde in PBS for 2 h at 4 °C. Control cells were not exposed to the recombinant IL-17A. The fixed cell culture samples were then immersed in 4% (wt/vol) sucrose in PBS for at least 24 h and were subsequently frozen in liquid nitrogen. For preembedding immunocytochemistry, the coverslips were blocked in low-fat milk powder for 30 min at RT. The anti-IL-17RA antibody was applied in PBS/0.2% (wt/vol) BSA and incubated overnight at 4 °C. After six rinses with PBS/0.2% (wt/vol) BSA, the coverslips were incubated overnight at 4 °C with ultrasmall gold-conjugated antimouse F(ab′)2 in PBS/0.2% (wt/vol) BSA. The coverslips were then rinsed five times with PBS/0.2% (wt/vol) BSA and two times with PBS and then fixed with 2.5% (vol/vol) glutaraldehyde in PBS for 2 h at 4 °C. Silver enhancement was then performed for 1.5 h in darkness. Coverslips were postfixed with 0.5% (wt/vol) OsO_4_ in PBS for 15 min and embedded in Epon resin. Ultrathin sections of the coverslips were cut and examined with electron microscopy (EM 902; Zeiss, Oberkochen, Germany). Apart from the omission of the primary antibody, control sections were treated the same way. No control section showed labeling with gold particles.

### Statistical analysis

Data were calculated as means ± standard error of the mean. Statistical significance between different conditions was assessed with the Student *t* test. A value of p<0.05 was considered significant.

## Results

### Interleukin-6, soluble form of interleukin-6R, and soluble glycoprotein 130 expression in human corneal epithelial cells exposed to either *Staphylococcus aureus* or *Pseudomonas aeruginosa* supernatants

**Figure 1:**
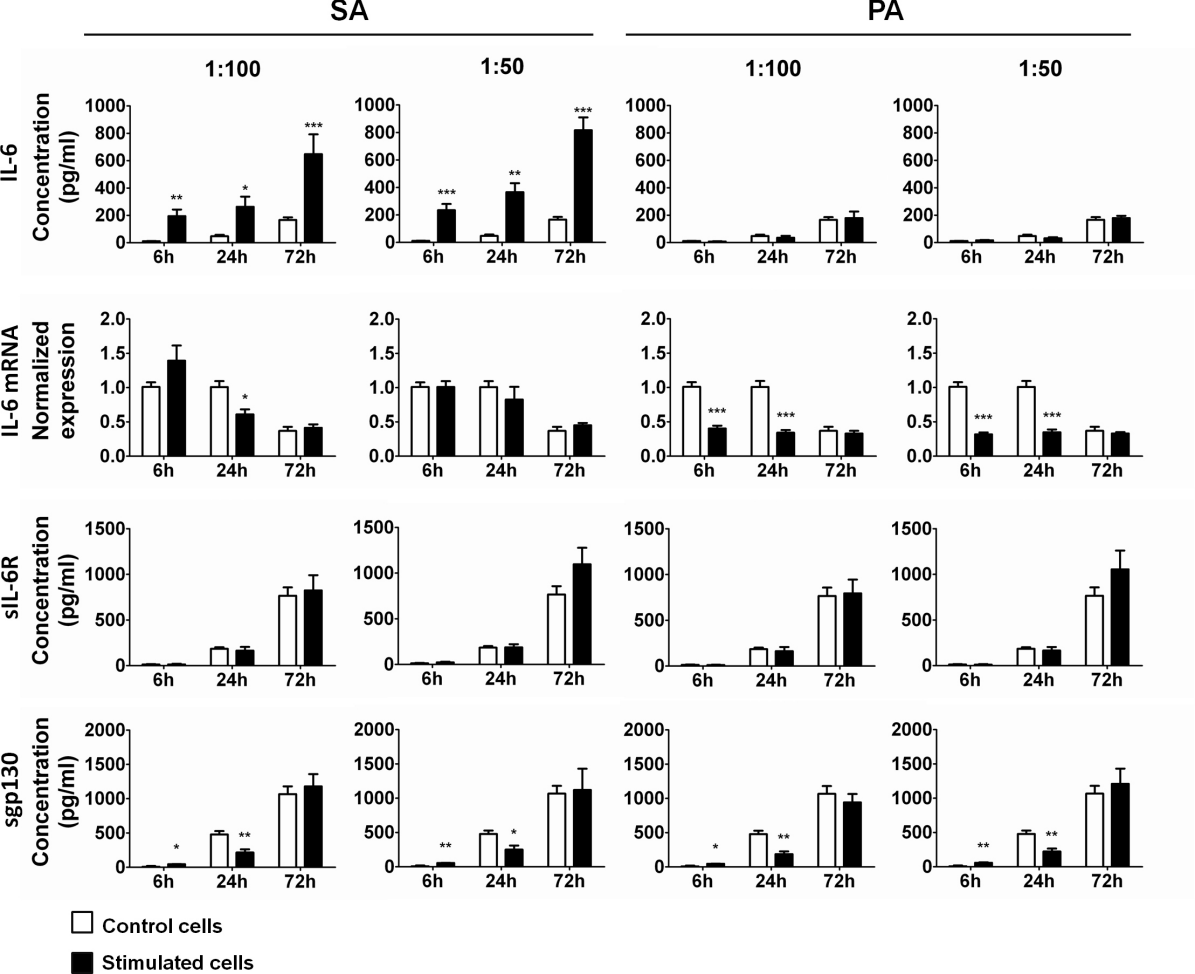
Interleukin 6, interleukin 6 Receptor, and soluble glycoprotein 130 secretion by HCE cells exposed to *Staphylococcus aureus* and *Pseudomonas aeruginosa* supernatants. Supernatants and mRNA from *Staphylococcus aureus* (SA)- and *Pseudomonas aeruginosa* (PA)-stimulated HCE cells (solid bars) and from control unstimulated cells (open bars) were collected at different time points. Bars represent the mean value of duplicates from three independent experiments. Values are expressed as mean±SEM, and statistical significance, when compared to control unstimulated cells, is indicated with asterisks (*p<0.05; **p<0.005; ***p<0.0005). Interleukin (IL-) 6, soluble IL-6 Receptor (sIL-6R), and soluble glycoprotein (sgp)130 were each secreted in a time-dependent manner. IL-6 secretion increased significantly in SA-stimulated cells but not in PA-stimulated cells. IL-6 mRNA expression was higher in control and SA-stimulated cells at 6 h and 24 h, but not at 72 h. In PA-stimulated cells, IL-6 mRNA levels were significantly lower than those of the controls at 6 h and 24 h. sIL-6R secretion did not change significantly under any condition; however, sgp130 secretion increased significantly in each condition at 6 h of stimulation and then significantly decreased at 24 h. By 72 h, there were no significant differences between the control and stimulated cells.

Potential inflammatory responses were evaluated in SA- and PA-stimulated HCE cells by analyzing changes in the expression of IL-6 and the principal molecules in the signaling pathway, such as sIL-6R and sgp130. Control unstimulated HCE cells secreted IL-6, sIL-6R, and sgp130 in a time-dependent manner (Figure 1). In cells exposed to SA supernatants, the IL-6 levels were significantly increased for both dilutions when compared to control cells at every time of study, reaching 21-, 7-, and fourfold increases for the 1:50 dilution at 6, 24, and 72 h, respectively (p<0.0005, p<0.005, and p<0.0005). There were no differences in IL-6 secretion between PA-stimulated and control unstimulated cells at any time of study. IL-6 mRNA expression in control cells unexposed to bacterial supernatants remained constant at 6 and 24 h, and decreased at 72 h (p<0.0005, Figure 1). In SA-stimulated cells, IL-6 mRNA expression tended to increase at earlier times and then drop to the control mRNA levels at 72 h of stimulation. However, in PA-stimulated cells, IL-6 mRNA expression was significantly decreased at 6 and 24 h, but by 72 h, the expression was similar to the control levels. There were no differences in sIL-6R levels between the control and stimulated cells, in any condition. However, sgp130 levels in response to both dilutions of SA and PA were significantly increased relative to the control cells at 6 h, were significantly decreased at 24 h, and were not different from the control cells at 72 h.

To determine if the changes in cytokine expression were due to a toxic effect of soluble pathogen-associated molecular patterns (PAMPs), we measured the viability of the HCE cells exposed to bacterial supernatants. The XTT-toxicity assay showed that the viability of cells exposed to both bacterial supernatants at both dilutions was higher than 90% at any time of study (data not shown).

### Interleukin-17RA expression in human ocular tissues

**Figure 2:**
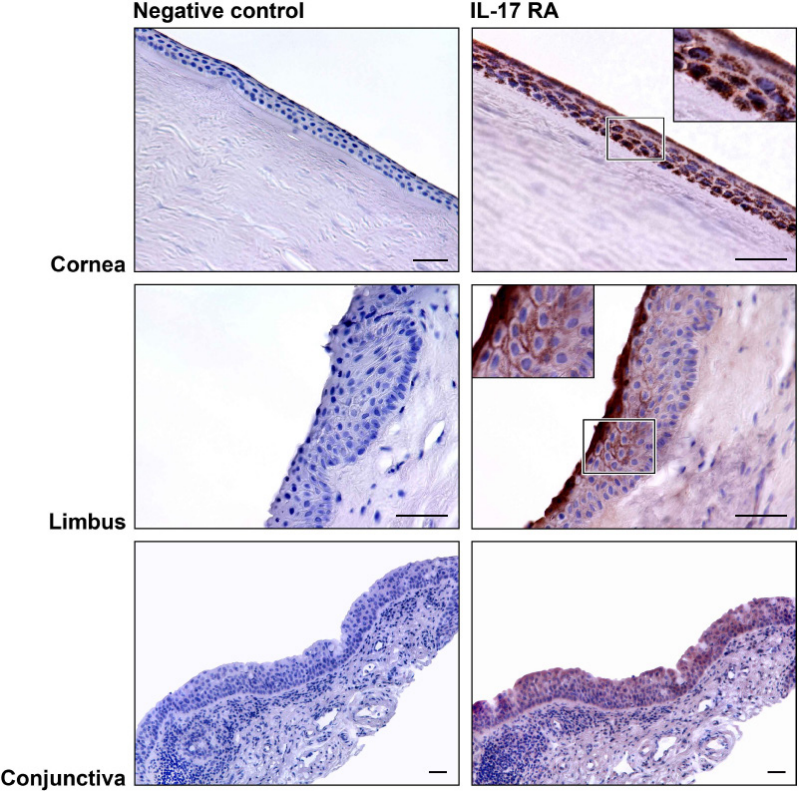
Interleukin 17 Receptor A expression in human ocular surface tissues. Ocular surface tissues (cornea, limbus, and conjunctiva) expressed histochemically detectable Interleukin 17 Receptor A (IL-17RA) as seen in these representative micrographs. The superficial layers were the most intensely stained, and the deeper cells had different patterns depending on the location: dotted staining in the cornea, staining in the cell-to-cell-contact regions in the limbus, and homogeneous staining in the conjunctiva. Insets show higher magnification of staining distribution. Bar=40 µm.

To our knowledge, IL-17RA has not been identified in epithelial cells from human ocular surface tissues, so we used immunohistochemistry to describe the distribution of IL-17RA in sections from cadaveric donors (Figure 2). The corneal epithelium had a granular perinuclear cytoplasmic reactivity in all epithelial layers, but it was more obvious in the superficial cell layer. At the limbus, the superficial epithelial cells also reacted strongly with the antibody. Deeper epithelial layers demonstrated reactivity only weakly at the cell surfaces. The basal cell layer showed no reactivity. The cytoplasm of conjunctival epithelial cells, but not goblet cells, was also positive for IL-17RA.

### Interleukin-17A and interleukin-17RA expression in human corneal epithelial cells exposed to either *Staphylococcus aureus* or *Pseudomonas aeruginosa* supernatants

**Figure 3:**
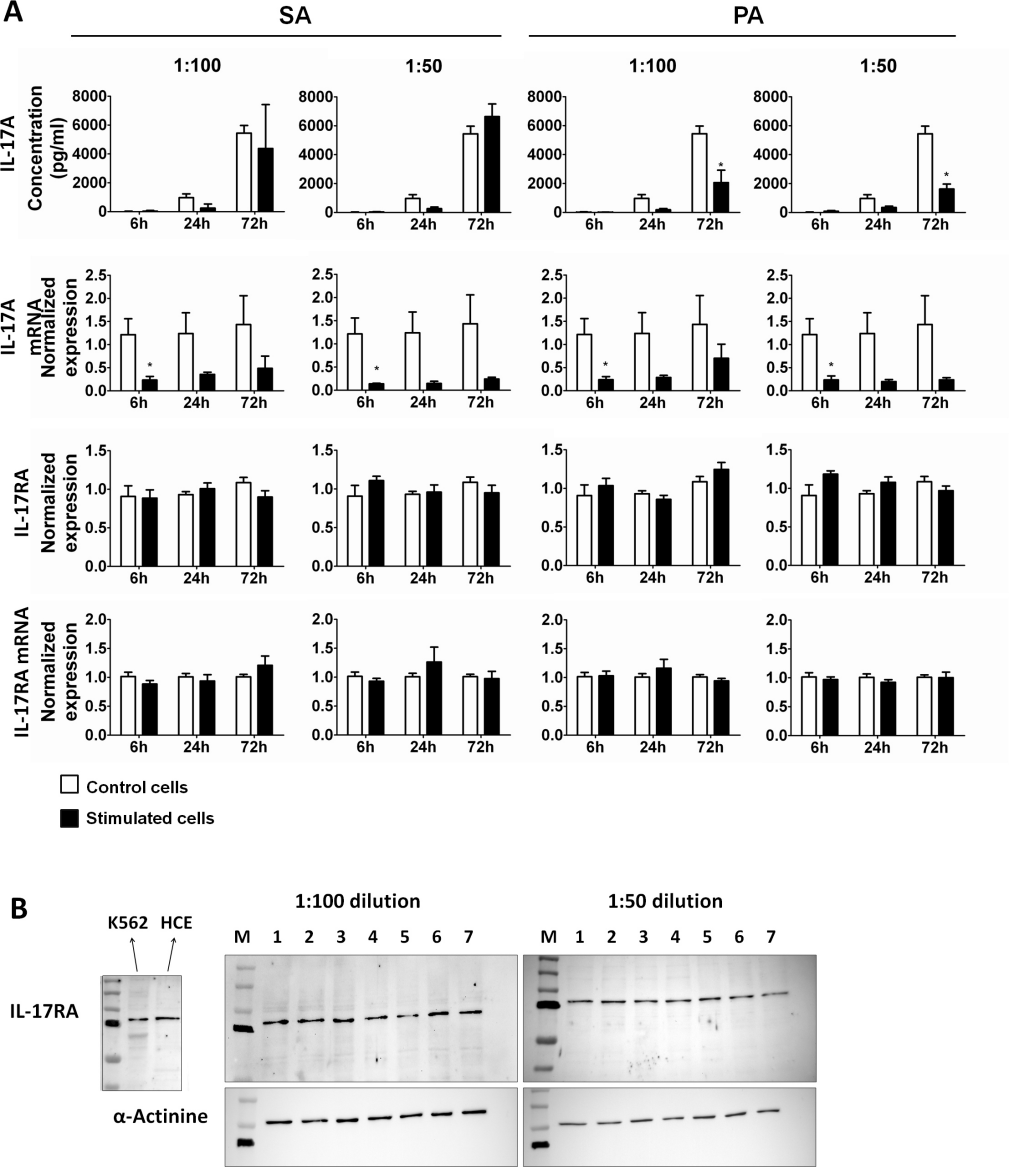
Interleukin 17A and Interleukin 17 Receptor A expression by HCE cells exposed to *Staphylococcus aureus* and *Pseudomonas aeruginosa* supernatants. **A**: Supernatants, protein, and mRNA from *Staphylococcus aureus* (SA)- and *Pseudomonas aeruginosa* (PA)-stimulated HCE cells (solid bars) and from control unstimulated cells (open bars) were collected at different time points. Bars represent the mean value of duplicates from three independent experiments. Values are expressed as mean±SEM, and statistical significance, when compared to control unstimulated cells, is indicated with asterisks (*p<0.05). Interleukin (IL-) 17A was secreted in a time-dependent manner that was not altered under any condition. IL-17A mRNA expression remained constant in the control unstimulated cells. In stimulated cells, it was significantly decreased at 6 h and tended to be lower at 24 h and 72 h than in the control cells. IL-17 Receptor A (IL-17RA) protein and mRNA expression did not change significantly with time. **B**: Internal control for specific band determination: K562 and HCE protein lysates, and representative western blot images for each condition: M- Molecular weight markers; 1- SA, 6 h; 2- PA, 6 h; 3- SA, 24 h; 4- PA, 24 h; 5- SA, 72 h; 6- PA, 72 h; 7- C, 72 h.

We determined whether HCE cells were involved in the Th17 response when stimulated with SA and PA supernatants by measuring IL-17A and IL-17RA protein and mRNA expression. Control unstimulated HCE cells secreted IL-17A to the culture medium in a time-dependent manner (Figure 3A). There were no differences in IL-17A secretion between the SA-stimulated and the control unstimulated cells at any time of study. In the cells exposed to the PA supernatants, there was a significant decrease in secreted IL-17A levels for both dilutions when compared to the control cells at 72 h (p<0.05). IL-17A mRNA expression in control cells unexposed to bacterial supernatants remained constant over time. Exposure to bacterial supernatants tended to decrease IL-17A mRNA expression; however, this was significant only at 6 h (p<0.05).

Western blot analyses were performed to quantify IL-17RA expression. An 85 kDa immunoreactive band was found in the control K562 cell lysates, unstimulated HCE cells, and bacterial supernatant-stimulated HCE cells (Figure 3B). IL-17RA expression in control cells was unchanged over time, and there were no differences between the control and stimulating conditions at any time of study. Moreover, there were no significant variations in IL-17RA mRNA expression in all control and stimulating conditions (Figure 3A).

**Figure 4:**
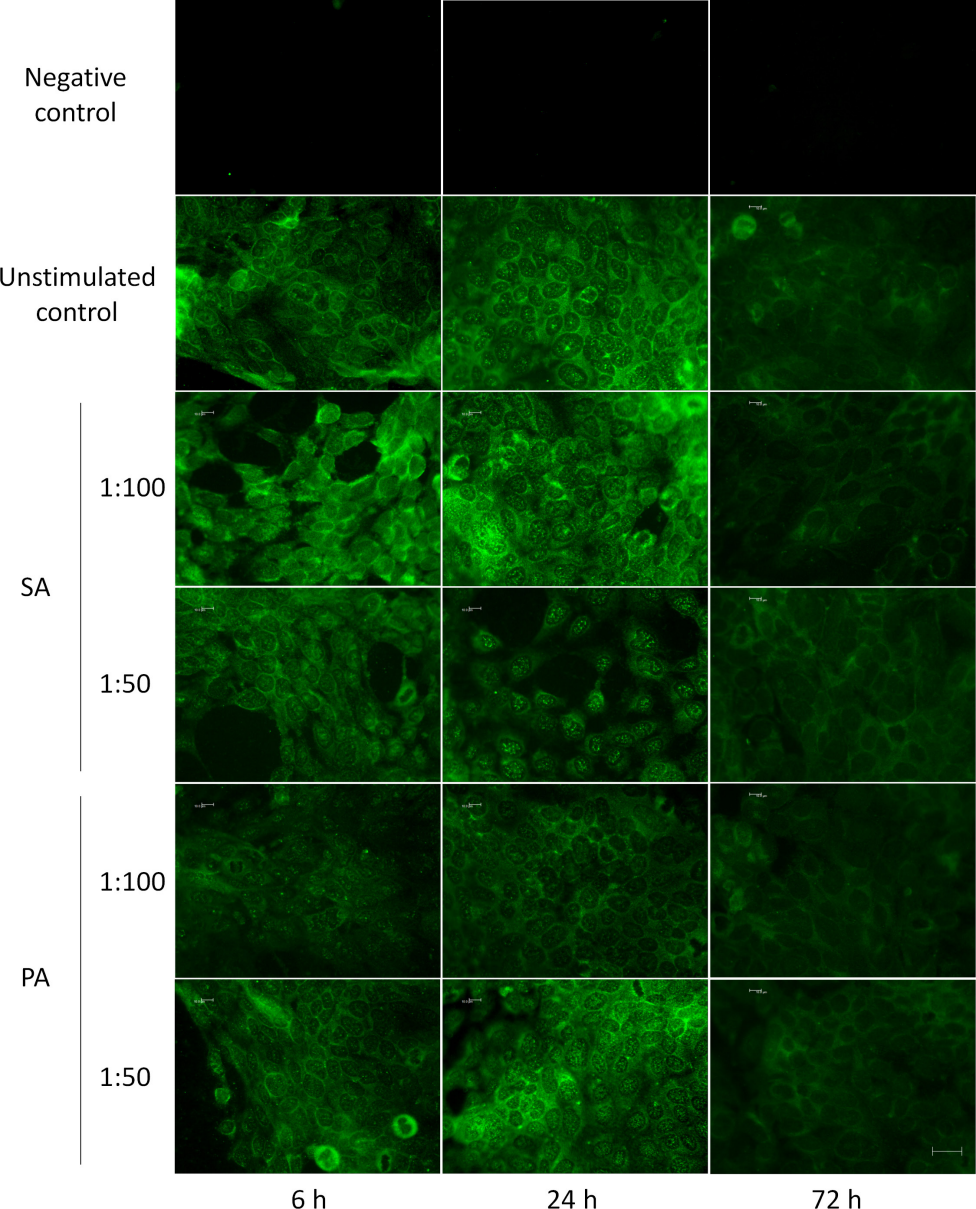
Interleukin 17 Receptor A immunofluorescence in HCE cells exposed to *Staphylococcus aureus* and *Pseudomonas aeruginosa* supernatants. Interleukin 17 Receptor A (IL-17RA) expression was analyzed with immunocytochemistry in fixed HCE cells exposed to *Staphylococcus aureus* (SA) and *Pseudomonas aeruginosa* (PA) supernatants at different dilutions and time points. Experiments were performed three times, and a representative image of each condition is shown. Bar=25 µm. Control unstimulated cells expressed IL-17RA in the cytosol, plasma membrane, and close to the nucleus. Stimulated cells also expressed IL-17RA.

IL-17RA was also identified in control HCE cells with immunofluorescence microscopy (Figure 4). HCE cells expressed IL-17RA throughout the culture period, showing vesicular reactivity close to the nucleus and a homogeneous reactivity in the cytosol and at the cell surfaces. At longer times of study, the reaction intensity seemed to slightly decrease. Fluorescence signal was also present in SA- and PA-stimulated cells, regardless of the stimulation dilution or time. Stimulation with bacterial supernatants did not modify the pattern or the signal intensity in any case, although fluorescence slightly decreased over time, particularly in the areas close to the nucleus.

### Interleukin-17RA expression in human corneal epithelial cells exposed to interleukin-17A

**Figure 5:**
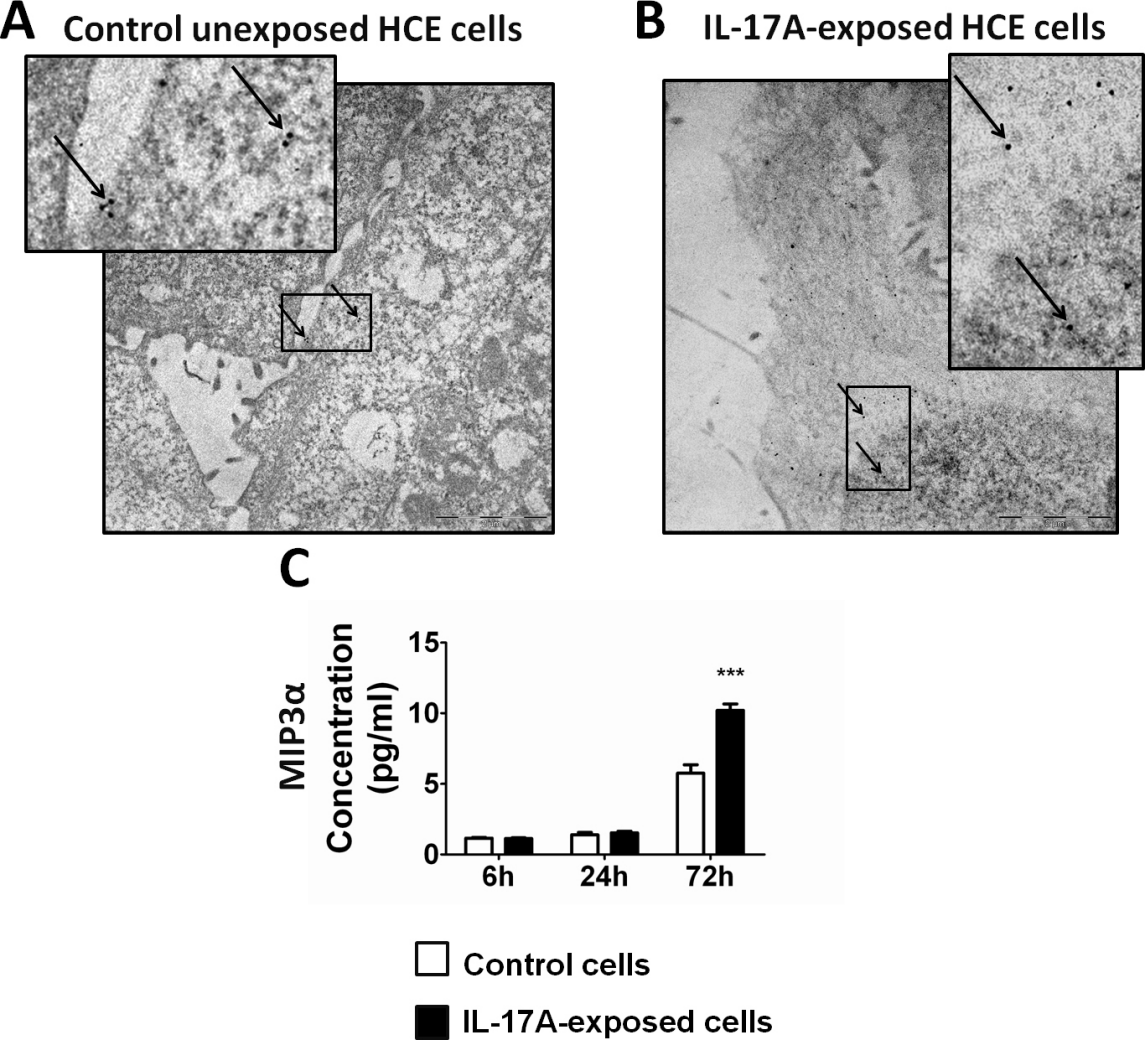
Interleukin 17 Receptor A expression and MIP3α secretion by HCE cells stimulated by interleukin 17 A. **A**, **B**: Interleukin 17 Receptor A (IL-17RA) expression was analyzed with immunogold labeling and electron microscopy in HCE cells either untreated or exposed to interleukin (IL-)17A, respectively. Control unexposed cells expressed IL-17RA in the cytosol. In IL-17A-exposed cells, immunogold labeled IL-17RA was present in the nucleus in addition to the cytosol. Bar=2 µm. Insets show higher magnification of gold-labeled antibody. **C**: After 72 h, there was a significant increase in MIP3α in supernatants from cells exposed to IL-17A (solid bars) compared to unexposed controls (open bars), indicating an active receptor for IL-17A. Bars represent the mean value of duplicates from three independent experiments. Values are expressed as mean±SEM, and statistical significance is indicated with an asterisk (p<0.05).

Possible changes in IL-17RA were evaluated in HCE cells exposed to IL-17A. IL-17RA was visualized with immunocytochemistry and immunogold-labeling electron microscopy in unexposed and exposed cultured HCE cells (Figure 5A, B). After exposure to recombinant IL-17A, more gold particles were visible than without exposure.

Western blot analyses were performed to quantify IL-17RA expression. An 85 kDa immunoreactive band was present in control and IL-17A-exposed cells. IL-17RA expression in IL-17A-exposed cells tended to slightly increase over time, but the differences were not statistically significant (data not shown). Additionally, IL-17RA mRNA expression did not vary significantly among all control and exposure conditions (data not shown).

To determine whether IL-17RA was a functional receptor, we measured the MIP3α levels in the cell culture supernatants from the control unexposed and the IL-17A-exposed HCE cells (Figure 5C). HCE cells secreted MIP3α in a time-dependent manner, and the expression was significantly enhanced by IL-17A exposure after 72 h (p<0.0005).

## Discussion

During infectious inflammatory processes, the immune system determines the T cell response (Th1, Th2, or Th17) depending on a broad variety of genetic and environmental factors. These factors include the type of cytokines secreted by the infected cells or specific immune cells at the place of infection. The Th17 pathway is commonly activated during infection with extracellular bacteria through cytokines, including IL-6, involved in differentiating naïve T cells into Th17 cells [36]. Tekstra et al. have reported that vascular endothelial cells can initiate and support an inflammatory response after infection by pathogenic microorganisms [37], but the ability of epithelial cells to achieve the same response has not completely been elucidated thus far. In our work, HCE cells under basal conditions secreted IL-6 into the culture medium in a time-dependent manner. Moreover, SA supernatant, but not PA supernatant, induced a significant increase in IL-6 secretion. This in vitro model of corneal bacterial infection demonstrates how HCE cells are able to react against SA-derived harmful products (PAMPs) by increasing IL-6 production. The increase in proinflammatory cytokines after PAMP stimulation could come from potential toxicity; however, the viability levels of the HCE cells exposed to bacterial supernatants remained close to that of the control cells, as determined with the XTT-based toxicity assay. It is possible that bacterial PAMPs lead to a nearly immediate increase of IL-6 secretion through mRNA stabilization, a characteristic mechanism of short-lived cytokines, as explained by Liton et al. [38]. At earlier stimulation time points, even before 6 h, IL-6 mRNA was rapidly translated into protein, contributing to the high increase in IL-6 secretion observed in SA-stimulated cells. In addition, Lutter et al. reported that bacterial agents restrict eukaryotic protein synthesis [39]. For IL-6, this leads to the inhibition of mRNA degradation. Thus, although there was no increase in mRNA production, we found an increase in IL-6 protein expression provoked by restricted mRNA degradation. Later, after 72 h, IL-6 mRNA levels dropped to the control levels while the secreted IL-6 progressively increased over time. Decreasing levels of IL-6 mRNA could indicate a transient effect of the in vitro stimulation with bacterial PAMPs. However, while PA-stimulation did not show any effect in secreted IL-6 expression, we observed a significant decrease in IL-6 mRNA expression at 6 h and 24 h. This could indicate regulation by the HCE cells, inhibiting IL-6 secretion, avoiding in this way a proinflammatory milieu when IL-6 is not actually needed. In our work, we used a PA strain that is perhaps not pathogenic or not pathogenic to HCE cells, and further studies are needed to determine whether other PA strains, including ocular pathogenic strains, produce a different response.

Desiccating stress, another common cause of ocular surface inflammation, increases IL-6 and IL-17A expression [20]. IL-17A is also expressed in corneas from patients with herpetic stromal keratitis [21]. However, our results show that the production of IL-17A, the hallmark protein of the Th17 pathway, is not increased after stimulation with the bacterial supernatants in HCE cultures. Furthermore, we observed a decrease in IL-17A mRNA levels at 6 h exposure. This leads us to think that corneal epithelial cells may not be the main sources of IL-17 during infectious inflammation, but that they are able to downregulate IL-17A mRNA production. Nevertheless, they can be considered active producers of IL-6. In the inflamed eye, IL-6 promotes the differentiation of the lymphocytes attracted to the Th17 cells [9]. The presence of these cells in the affected area would explain an increase in IL-17A amounts at the ocular surface. Moreover, potential innate immune cells present at the site of infection may respond to bacterial products by producing IL-17A. In turn, this could act via IL-17R on epithelial cells, resulting in secretion of chemotactic factors such as MIP-3α that may recruit more leukocytes to the site of infection. This demonstrates a protective role of IL-17A when expressed in response to bacterial infection, a role that is different from the pathogenic role when expressed by Th17 cells in autoimmune or chronic inflammatory diseases [17].

**Figure 6:**
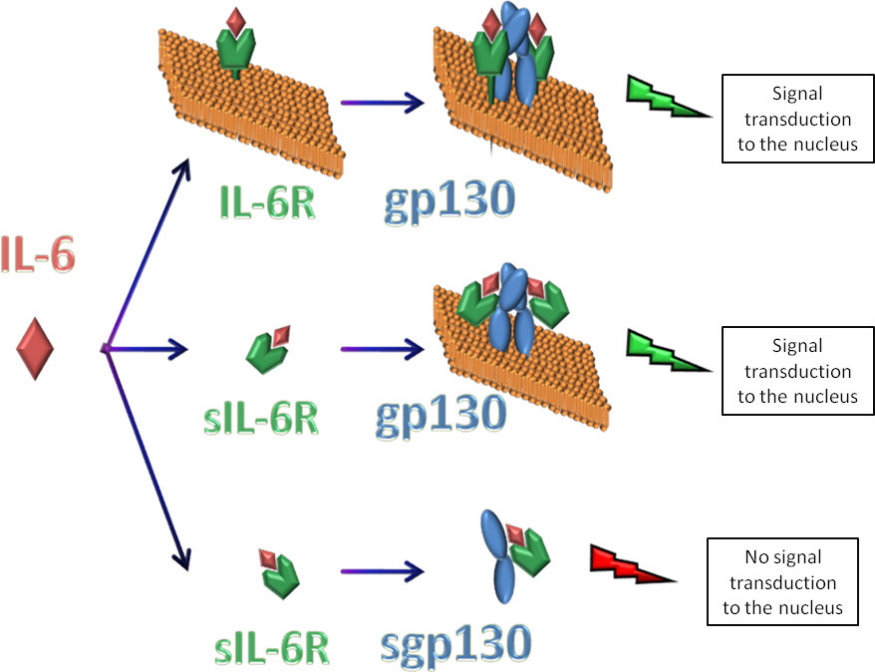
Possible interactions between interleukin 6 and its receptor complex, and regulation by soluble glycoprotein 130. Interleukin 6 Receptor can be present as both a transmembrane (IL-6R) and a soluble protein (sIL-6R). Glycoprotein (gp) 130 can be present linked to cell membranes and as a soluble fragment (sgp130). Interleukin (IL-) 6 can bind either IL-6R or sIL-6R, and both receptors are able to initiate the IL-6 signaling cascade by interacting as a homodimer with two copies of transmembrane gp130. However, sgp130 acts as an antagonist by binding sIL-6R/IL-6, inhibiting their interaction with transmembrane gp130, and blocking the IL-6 signaling pathway.

For understanding and treatment of inflammatory diseases, components of the signaling pathway are sometimes as crucial as the ligands themselves. Therefore, we also studied soluble IL-6R and gp130, both important to the IL-6 signaling pathway. Although IL-6 expression was increased in the presence of bacterial supernatants, sIL-6R secretion by the HCE cells was not significantly increased at any of the studied stimulation times. There was a tendency to increase sIL-6R secretion in 1:50 dilution-stimulated cells as compared to the control. Taking into account that IL-6R expression is mainly limited to hepatocytes and some leukocytes, our results may point out that other cell types at the ocular surface are responsible for the secretion of the soluble form of the receptor to assure the propagation of IL-6 signals into the infected corneal epithelial cells, at least at earlier time points of infection. However, expression of sgp130 increased significantly at the earliest time point and then decreased at the next time point. At later times, there were no detectable differences between the control and treated cells. The soluble receptor sgp130 acts as an antagonist by inhibiting the binding of the preassembled IL-6-sIL-6R to the transmembrane gp130, thus effectively restraining IL-6 signaling (Figure 6) [40]. This may reflect changes in response to increased IL-6 secretion. Thus, the initial protective response to neutralize potential inflammatory effects of IL-6 would be later overcome by the reduced sgp130 levels. This could result in a more proinflammatory outcome in response to bacterial products. Therefore, based on our results, we assume that the same HCE cells that are producing high amounts of the proinflammatory cytokine IL-6 are also autoregulating its production by secreting a potential inhibitor, the sgp130 receptor.

Mouse corneal fibroblasts express IL-17RA [41], and IL-17RA mRNA has been found in human corneal fibroblasts [21]. Until now, IL-17RA protein expression has not been identified in cells of the human ocular surface, but we found IL-17RA protein expressed in human corneal, limbus, and conjunctival epithelial cells. A different pattern of reactivity with the antibody was present depending on the cell type and its location. Considering the distribution of the reaction, corneal and limbal sections presented intense reactivity in the most apical epithelial cell layer. However, expression of IL-17RA in the conjunctival epithelium was present as weaker and more homogeneous reactivity in every cell layer, even in stromal cells. The superficial cells of the ocular surface are usually the most exposed to pathogens, and this could explain the abundant presence of cytokine receptors in the most superficial epithelial cell layers of corneal and limbal cells, but not in the conjunctiva, which is not directly involved in vision.

We also found differences among the three different regions regarding the pattern of the reaction within the cells. IL-17RA expression was present as perinuclear vesicular reactivity and in the cytosol of corneal epithelial cells, which is in agreement with our in vitro results of immunofluorescence. Limbal cells were stained only at the cell-cell contact regions, and conjunctival epithelial cells presented homogeneous reactivity. We can presume that limbal cells are in an active region in terms of IL-17A signaling, which would explain the reactivity on cell membranes, and paracrine signaling in the IL-17 cascade. The presence of the receptor in granules within the cytosol of corneal epithelial cells suggests that IL-17RA is present as a precursor awaiting the appropriate signal that triggers IL-17RA processing.

N-glycosylation of IL-17RA has been reported in T cells [42] as a mechanism of receptor maturation. A glycosylated 110 kDa protein is described in patients with oral inflammation [43]. In our work, IL-17RA was found as an 85 kDa protein, which is lower than the predicted molecular weight based on amino acid sequence analysis (96 kDa; UniProt Knowledgebase). We first thought that this could correspond to an immature form of the receptor and that the stimulation with bacterial supernatants did not lead to the receptor’s maturation. Thus, we determined whether IL-17RA maturation could occur in the presence of higher amounts of the main ligand IL-17A. The stimulation of HCE cell cultures with IL-17A did not lead to a higher molecular weight protein as determined with SDS–PAGE and western blotting. However, our MIP3α secretion results in IL-17A-stimulated HCE cells indicate that the IL-17RA was functional, as it was able to transduce a signal, leading to an increase in MIP3α expression [30].

IL-17RA is needed for the signal transmission of IL-17, the main effector cytokine of the Th17 pathway, which is the usual response for extracellular pathogens. IL-17RA-deficient mice are vulnerable to infections caused by extracellular pathogens [17], though no data concerning the cornea are available. Although SA and PA are extracellular pathogens, in our work, corneal epithelial cells were not stimulated to express higher levels of IL-17RA protein or mRNA by bacterial PAMPs. This is probably because corneal epithelial cells produce a constant amount of mRNA for IL-17RA, keeping a stable level, in case the receptor is needed for translation into protein after a challenge. Additionally, one must take into account that we used only PAMPs, not real bacteria. Thus, an in vivo approach is needed to have a full understanding of what occurs during infection.

In conclusion, corneal epithelial cells of the HCE cell line are not innate sources of IL-17 during exposure to bacterial supernatants in vitro. However, these cells may act as indirect participants in the Th17 pathway by secreting IL-6, controlling its signal propagation with the secretion of sgp130, and expressing IL-17RA, the main receptor of the IL-17A propagation signal. Further studies in human primary epithelial cells are warranted to clarify the regulation of this signaling pathway.
